# Thirteen -weeks ovarian pregnancy following in vitro fertilization for primary infertility treatment: A case report

**Published:** 2014-11

**Authors:** Tahereh Ashrafganjoei, Behzad Nemati Honar, Sara Defaee

**Affiliations:** 1 *Department of Obstetrics and Gynecology, Imam Hussein Hospital, Shahid Beheshti University of Medical Sciences, Tehran, Iran.*; 2 *Department of Surgery, Imam Hussein Hospital, Shahid Beheshti University of Medical Sciences, Tehran, Iran.*

**Keywords:** *Ectopic pregnancy*, *Ovarian pregnancy*, *In vitro fertilization*

## Abstract

**Background:**

Ovarian pregnancy constitutes 0.15-3% of all ectopic pregnancies. The incidence of ectopic pregnancy is on the rise owing to evolution in assisted reproductive techniques (ART). The incidence reported following In vitro fertilization (IVF) or embryo transfer (ET) is 0.27% per clinical pregnancy.

**Case: **We present a case of a 13-weeks ovarian pregnancy following IVF-ET and through a review of the literature, the specific symptomatology, diagnostic criteria, and treatment of this particular pathology will be described.

**Conclusion:**

Ovarian pregnancy is a rare condition and its diagnosis is difficult and relies on criteria based on intraoperative and histopathological findings. The management is, in spite of medical improvement, based on surgery. But the trend has shifted towards conservative surgeries in majority of cases.

## Introduction

Ectopic pregnancy is an important health problem and accounts for 10% of all maternal mortality. Ovarian pregnancy constitutes 0.15-3% of all ectopic pregnancies and the incidence reported following In vitro fertilization (IVF) or Embryo Transfer (ET) is 0.27% per clinical pregnancy (1). Ovarian pregnancy can be classified as primary and secondary. It is called as primary when the ovum is fertilized while it is still within the follicle and called as secondary when the fertilization takes place in the tube and when the concept is later regurgitated and implanted in the ovarian stroma (1).

“Primary ovarian pregnancy is one of the rarest types of extra uterine pregnancy. The cause of primary ovarian pregnancy remains obscure and it would seem to be secondary to reflux of the fertilized oocyte to the ovary” (2). “The conditions which are most commonly confused with ovarian pregnancy, both clinically and pathologically, are a ruptured hemorrhagic corpus luteal cyst, “chocolate’’ cysts and a ruptured distal tubal ectopic pregnancy” (3). Diagnosis of ovarian pregnancy should be suspected from elevated βHCG, lack of intrauterine gestation, a complex ovarian mass on USG, patient’s risk factors, in addition to the Spiegelberg′s criteria (2). 

Spiegelberg suggested four criteria to distinguish a primary ovarian pregnancy from a distal tubal pregnancy which secondarily involved the ovary. They are: 1) the fallopian tube with its fimbriae should be intact and separate from the ovary 2) the gestational sac should occupy the normal position of the ovary 3) the gestational sac should be connected to the uterus by the ovarian ligament 4) a histologically proven ovarian tissue should be located in the sac wall (1).

Ultrasound, especially transvaginal ultrasonography, has proven to be a valuable tool in its diagnosis, where the hyper echoic appearance of villosity which is surrounded by thickened hypo echoic ovarian tissue is the only indication of an ovarian ectopic gestation but the diagnosis is unclear and could be missed in many cases (4). Ovarian pregnancies usually terminate in rupture during the first trimester in 91% of the cases, in 5.3% cases in the second trimester and in 3.7% cases in the third trimester (5). 

Recently, there has been an increase in the incidence of ovarian pregnancies due to better diagnostic modalities such as transvaginal ultrasonography and because of wider use of intrauterine contraceptive device (IUCD), ovulatory drugs, assisted reproductive techniques such as in IVF or ET, but Primary ovarian pregnancy may occur without any classical antecedent risk factors (2, 6). 

Here, we report a case of primary ovarian pregnancy following IVF for primary infertility treatment.

## Case report

A 32 years old primigravid woman was referred to our hospital in 2013 with a probable diagnosis of ectopic pregnancy. She was at 12w of gestation according to her last menstrual period date. She had a 13 years history of primary infertility due to tubal factor. Her menstrual cycles were regular with normal hormonal profile. In her past medical history she had a laparoscopic left salpingectomy 4 years ago (because of left severe hydrosalpynx) and laparoscopic left ovarian cystectomy 3 years ago. Also, she had two cycles of IVF in 2010 and 2011 with negative outcomes.

Finally, the patient was candidate for ovulation induction and received GNRH antagonist. After the ovaries were punctured, total of 25 oocytes, 15 from left ovary and 10 from the right one were retrieved. Of 13 embryos, 4 four-celled embryos were transferred and 9 were freeze. Two weeks after ET, βhCG was negative. 3 weeks after ET, she had vaginal bleeding for 5 days with normal amount, but after this period, spotting for 30 days continued and then she had no bleeding for 35 days later. Also she had no coitus for 2 weeks before induction of ovulation, and during 8 weeks after ET (because of vaginal bleeding). So, she did a pregnancy test and βhCG level was 22,500 mIU/ml at day 90 after ET. The ultrasound study showed ectopic pregnancy in right adnexa, which was 13 weeks for gestational age with normal fetal heart rate; therefore she had been referred to our medical center for more evaluation and treatment.

At first visit, her general condition was good. She had no complaint of dizziness, shoulder pain, abdominal pain, nausea, vomiting or abnormal vaginal bleeding. Vital signs were normal (with a blood pressure of 110/60mm Hg and pulse rate of 84/min). Her abdomen was nontender, nondistended, and without any obvious palpable masses. The pelvic examination revealed normal cervix without any motion tenderness, vaginal bleeding or discharge. Left adnexal region was normal while there was a tender mass about 11 weeks in right adnexal region undetectable from uterus. Hematocrit was 36%.

**Figure 1:**
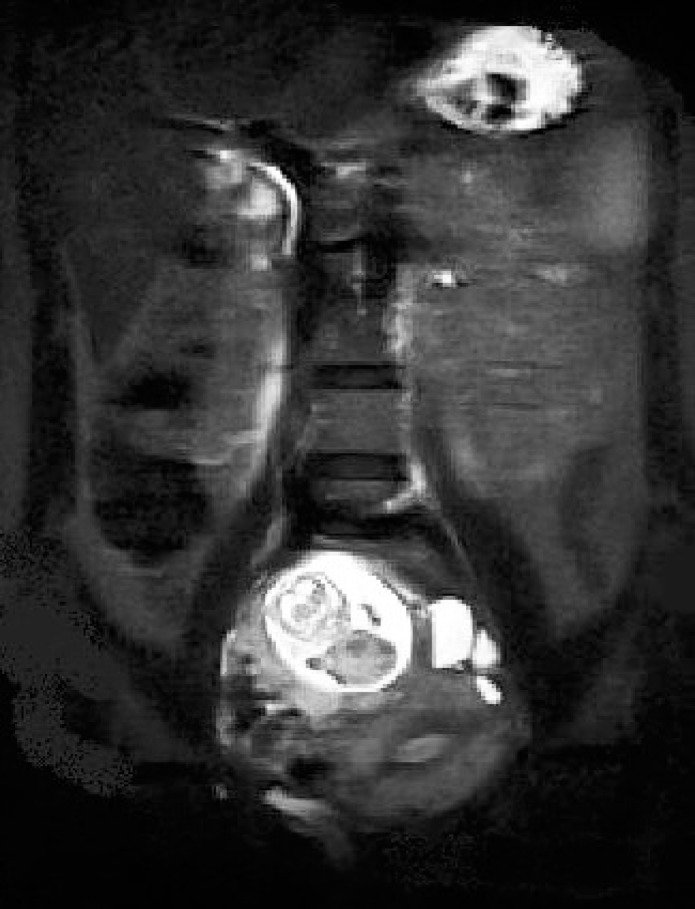
AbdominoPelvic MRI showing a large mass containing fetus in right adnexa in close contact with uterus.

**Figure 2:**
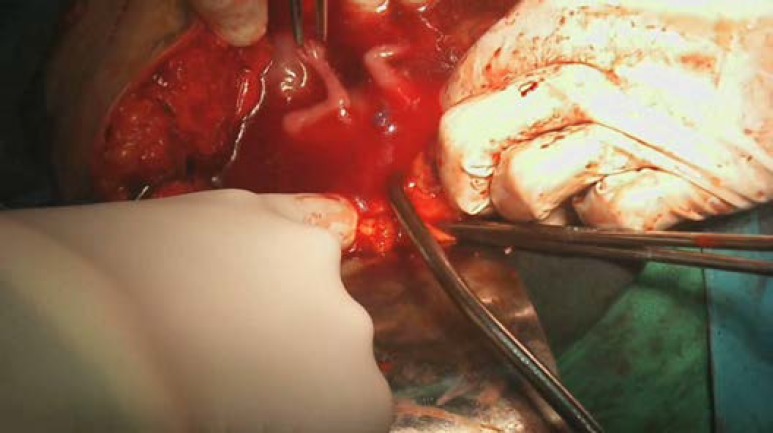
Clinical photograph showing gestational sac surrounded by right ovarian tissue and right tube.

The abdominal ultrasound reported a gestational sac, an alive fetus with gestational age of about 13 weeks. The sac was located beside the fundus of uterus and extended to the right adnexa. A normal placenta was seen in right lateral pelvic wall. Some fluid was seen in the peritoneal cavity. The uterus had normal size and its cavity was empty. In Doppler evaluation, arcuate vessels were seen between the sac and uterus that was suggestive for an ectopic pregnancy. An abdomino- pelvic MRI with and without contrast was done for diagnostic confirmation. It showed a large mass (12×10 cm) containing fetus with normal heart beat in right adnexa in close contact with uterine fundus. Placenta was located on inferolateral surface of this mass. Uterus was large with thick endometrium; abdominal cavity was unremarkable (Figure 1). With impression of advanced ectopic pregnancy, laparotomy was performed. Pelvic exploration revealed a normal uterus. Left fallopian tube was not observed (because of the previous left salpingectomy).The left ovary was normal, while the right ovary was enlarged with oozing of blood from the surface of a 10×12 cm hemorrhagic mass (Figure 2). 

**Figure 3:**
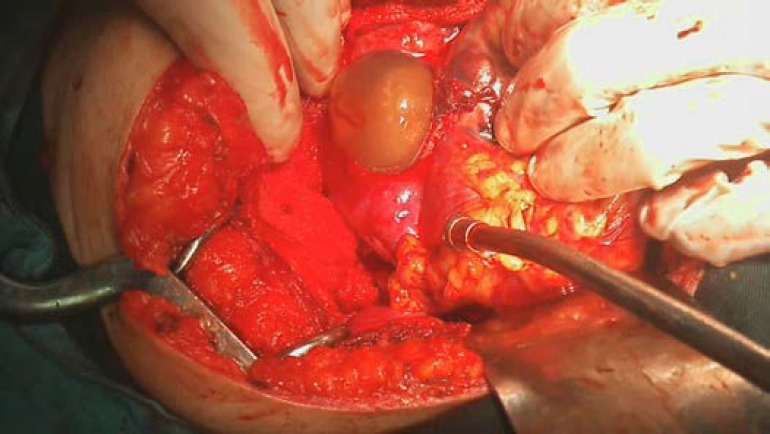
Clinical photograph showing the fetus is extracting from gestational sac.

Normal right tube was extended on the ovarian mass. Blood in the pouch of Douglas was observed. After releasing the omental adhesions, the mass was ruptured spontaneously and the sac and fetus were extracted (Figure 3), right salpango-ophorectomy was done but removal of the placenta (that was inserted in the inferior side of mass) caused to active bleeding that could not be controlled by stitches and foam gels; so the hemostasis was completed by inserting some long gases and a drain in posterior cul-de-sac. The estimated blood loss during the operation was about 2000 cc. The patient received 3 units of packed cells and referred to ICU. 

**Figure 4:**
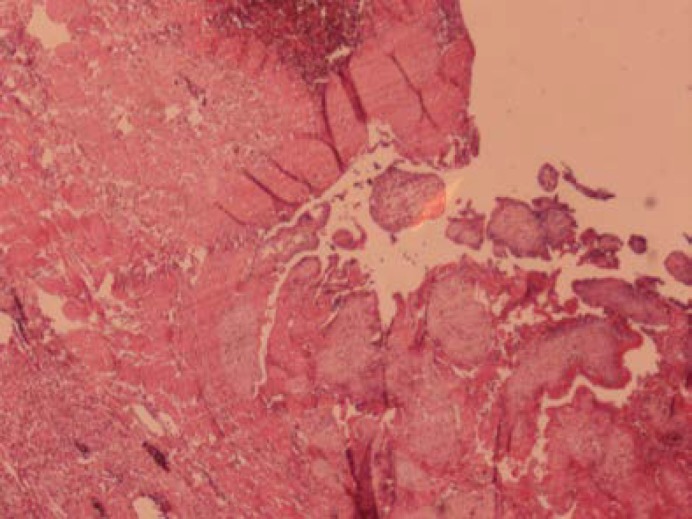
Right ovary showing ectopic pregnancy with villi (arrow), surrounded by ovarian tissue, hemorrhage and fibrin (H&E×100).

After 2 days she underwent re-laparotomy for extracting the long gases. She was discharged 4 days after the last operation. βhCG levels were measured several times and finally it was normal at day 25 after operation. Pathologic evaluation confirmed a right ovarian gestation, demonstrating ovarian tissue with focuses of hemorrhage and chorionic villi which fulfilling the four Spiegelberg′s criteria (Figure 4). 

## Discussion

Ovarian pregnancy is a rare condition and its diagnosis is difficult and relies on criteria based on intraoperative findings. There are recorded cases in which ovarian pregnancies have resulted in term delivery, and a few infants have survived (7). But the mean gestational age in some literatures is 45 days (8). The cause of ovarian pregnancy remains obscure. According to literatures, there are several mechanisms that tend to ectopic pregnancy after IVF or ET. The cases of ovarian pregnancy after IVF reported in the literature support the theory of reflux (2). Fertilization of the ovum inside the ovary or implantation of the fertilized ovum in the ovary seems to be most responsible causes in the etiology and pathogenesis of ovarian ectopic pregnancy (3).

Reverse migration of the embryo after deep deposition in the uterine cavity, use of large volume of culture fluid, presence of tubal pathology and pelvic inflammatory disease have been postulated as underlying factors (9). Other suggested causes are interference in the release of the ovum from the ruptured follicle, malfunction of the tubes and inflammatory thickening of the tunica albuginea. In this patient, tubal malfunction was known as the cause of infertility.

In our case, four embryos had been transferred. One of the most likely probabilities is reverse migration of one of these embryos toward the fallopian tube and implantation in the ovary that may be the result of the volume and pressure of culture injected during embryo transfer (10). Head tilt down position following ET predisposes the reverse migration of embryos (11). “Knutzen introduced the reverse migration concept by observing that radiopaque dye could enter the fallopian tubes in 38.2% of the patients easily after ET” (11). 

In this patient, ET was performed in supine instead of tilted position. Difficult ET may be another factor that stimulates the junction zone contractions which increases the risk of ectopic pregnancy. Lesny *et al* showed that a difficult ET stimulates junction zone contractions. Strong endometrial waves in the fundal area of uterus could move mock embryos into the fallopian tubes (11). In this case, the procedure was reported" Easy". Manipulation with tissue forceps, for the purpose of facilitating ET, could affect uterine contractility but any manipulations hadn’t been used in this case during ET (11).

The management of primary ovarian pregnancy remains surgical therapy in the first step of diagnosis, Endoscopic surgery is now regarded as the standard surgical management of ectopic pregnancy (Royal College of Obstetricians and Gynecologists), however, in only a few case series operative laparoscopy has been used as the exclusive treatment for women with ovarian pregnancy and in most case reports open surgery is still used routinely despite the benefits of the minimal access approach (8). Little evidence is available in the literature about medical treatment with methotrexate, probably because ovarian pregnancy is diagnosed in emergency settings when surgical treatment represents the gold standard (1). 

Methotrexate has been used successfully to treat unruptured ovarian pregnancies (12-14). If the patient desires a future pregnancy, the preservation of ovaries is the most goal of the treatment. Now, with ultrasonography advances; it can be diagnosed early, leading to conservative treatment and preservative surgery. Although "The difficulty in making a definitive diagnosis of ovarian pregnancy on ultrasonography limits the use of medical treatment as laparoscopy is usually needed for diagnosis" (8). 

Ovarian pregnancy had been treated by ipsilateral oophorectomy, but the trend has since shifted towards conservative surgeries such as cystectomy or wedge resection, which are performed during either laparotomy or laparoscopy (1). In our case, we did not use methotrexate because the advanced gestational age and the large size of placenta would probably result in medical treatment failure. Also we could not preserve the right ovary and fallopian tube because of the advanced pregnancy, severe placental adhesions to pelvic floor and active bleeding after extraction of conception products. Furthermore, the patient has the opportunity to conceive later through ART modalities. 

## References

[1] Farah Ziyauddin TK, Dalia Rafat, Meher Aziz, Nazima Haider (2012). A Case Report: Primary Ovarian Pregnancy with a Contra lateral Ruptured Corpus Luteum: A Case Report. J Clin Diagnos Res.

[2] Scutiero G, Digioria p, Spada A, Greco P (2012). Primary Ovarian Pregnancy and Its Management. JSLS.

[3] Ramachandran A, Sharma S, Pratap K, Rajesh B, Akhila V, Ramayapally A (2012). Ovarian Pregnancy following Intracytoplasmic Sperm Injection and Embryo Transfer: A Case Report. Case Rep Obstet Gynecol.

[4] Bouyer J, Coste J, Fernandez H, Pouly JL, Job-Spira N (2002). The sites of an ectopic pregnancy: A 10 year population based study of 1800 cases. Hum Reprod.

[5] Das S, Kalyani R, Lakshmi V, Harendra Kumar ML (2008). Ovarian pregnancy. Indian J Pathol Microbiol.

[6] Kissler S, Wiegrat I, Kohl J, Rody A, Gaetje R, Kaufmann M (2006). Repeated ectopic pregnancy after ICSI therapy and embryo transfer:a case report and literature review. J Reproduktionsmedizin und Endokrinologie.

[7] Gary Cunningham F, Kenneth JL, Steven LB (2010). Williams Obstetrics.

[8] Odejinmi F, Rizzuto MI, MacRae R, Olowu O, Hussain M (2009). Diagnosis and Laparoscopic Management of 12 Consecutive Cases of Ovarian Pregnancy and Review of Literature. J Minim Invas Gynecol.

[9] Priya S, Kamala S, Gunjan S (2009). Two interesting cases of ovarian pregnancy after invitro fertilization-embryo transfer and its successful laparoscopic management. Fertil Steril.

[10] Pope CS, Cook EKD, Arny M, Novak A, Grow DR (2004). Influence of embryo transfer depth on in vitro fertilization and embryo transfer outcomes. Fertil Steril.

[11] Knutzen V, Stratton CJ, Sher G, McNamee PI, Huang TT, Soto-Albors C (1992). Mock embryo transfer in early luteal phase, the cycle before in vitro fertilization and embryo transfer: a descriptive study. Fertil Steril.

[12] Chelmow D, Gates E, Penzias AS (1994). Laparoscopic diagnosis and methotrexate treatment of an ovarian pregnancy: a case report. Fertil Steril.

[13] Raziel A, Golan A (1993). Primary ovarian pregnancy successfully treated with methotrexate. Am J Obstet Gynecol.

[14] Shamma FN, Schwartz LB (1992). Primary ovarian pregnancy successfully treated with methotrexate. Am J Obstet Gynecol.

